# The Marsupial Database: A comprehensive dataset on the ecology and life history of American and Australasian marsupials

**DOI:** 10.1002/ecy.70358

**Published:** 2026-03-19

**Authors:** Mariana Silva Ferreira, Rodrigo C. Rossi, Stephane Batista, Kathleen Sena, Kevin R. Bairos‐Novak, Shamia Farhana Shoma, Natália O. Leiner, Diego Astúa, Graeme Coulson, Diana O. Fisher, Natasha D. Harrison, Julian Nicholas Garcia Willmer, Giulia R. Silva, Gabriel Cupolillo, Marcus Vinícius Vieira, Emerson Monteiro Vieira, Thomas Püttker, Marcos de Souza Lima Figueiredo, Christopher R. Dickman

**Affiliations:** ^1^ Instituto de Biodiversidade e Sustentabilidade, Universidade Federal do Rio de Janeiro Macaé Rio de Janeiro Brazil; ^2^ Programa de Pós‐Graduação em Ecologia, Departamento de Ecologia, Instituto de Biologia, Universidade Federal do Rio de Janeiro Rio de Janeiro Rio de Janeiro Brazil; ^3^ Instituto de Biologia, Universidade Federal de Uberlândia Uberlândia Minas Gerais Brazil; ^4^ Queensland University of Technology Brisbane Queensland Australia; ^5^ School of the Environment University of Queensland Brisbane Queensland Australia; ^6^ Departamento de Zoologia Universidade Federal de Pernambuco Recife Pernambuco Brazil; ^7^ School of BioSciences University of Melbourne Melbourne Victoria Australia; ^8^ School of Biological Sciences University of Western Australia Perth Western Australia Australia; ^9^ School of Geography Earth and Environmental Sciences University of Birmingham Birmingham UK; ^10^ Instituto de Biologia, Universidade de Brasília Brasília Distrito Federal Brazil; ^11^ Programa de Pós‐Graduação em Biodiversidade e Biologia Evolutiva, Instituto de Biologia, Universidade Federal do Rio de Janeiro Rio de Janeiro Rio de Janeiro Brazil; ^12^ Departamento de Ciências Ambientais Instituto de Ciências Ambientais, Químicas e Farmacêuticas, Universidade Federal de São Paulo Diadema Brazil; ^13^ Departamento de Ecologia e Recursos Marinhos Universidade Federal do Estado do Rio de Janeiro Rio de Janeiro Rio de Janeiro Brazil; ^14^ School of Life and Environmental Sciences The University of Sydney Sydney New South Wales Australia

**Keywords:** body mass, comparative analyses, diet, macroecology, mammal, phenology, physiology, population density, reproduction, survival, trait, vertebrate

## Abstract

Marsupials are an important but typically neglected group of mammals that have been overlooked in many comparative analyses of vertebrate ecology and life history evolution. In order to address this knowledge bias, we have developed The Marsupial Database. The Marsupial Database contains traits for a phylogenetically diverse set of 414 extant and recently extinct (last 200 years) species from all seven modern marsupial orders (Dasyuromorphia, Didelphimorphia, Diprotodontia, Microbiotheria, Notoryctemorphia, Paucituberculata, Peramelemorphia) native to 34 countries in the Americas and Australasia. The database comprises 11,054 records of 35 traits describing anatomical, physiological, phenological, and reproductive characteristics, as well as information on species' ecology and current conservation status. Data were collected from 41 sources, comprising published databases and other relevant sources of information. By providing a centralized repository of marsupial ecological and life history traits, The Marsupial Database facilitates analyses of ecological and evolutionary patterns within the group, and also encourages inclusion of marsupials in comparative studies of mammals, vertebrates, and the entire animal kingdom. The Marsupial Database is free from copyright or proprietary restrictions. Please cite this data paper when using the data in publications or scientific presentations.

## CONFLICT OF INTEREST STATEMENT

The authors declare no conflicts of interest.

## Supporting information


Data S1.

## Data Availability

Data are available in the Supporting Information in Data S1. Data are also available in Figshare at https://doi.org/10.6084/m9.figshare.29626664.

