# Supplementary material for: The Marsupial Database: A comprehensive dataset on the ecology and life history of American and Australasian marsupials
Source: Ecology. 2026 Mar 19;107(3):e70358. doi: 10.1002/ecy.70358 (PMC13002554; doi:10.1002/ecy.70358)
Supplement: Supplementary file 1 — Data S1. [file ECY-107-e70358-s001.zip › Metadata_S1.pdf]

Metadata S1

The Marsupial Database: A comprehensive dataset on the ecology and life history of American and Australasian marsupials

Mariana Silva Ferreira, Rodrigo C. Rossi, Stephane Batista, Kathleen Sena, Kevin R. Bairos-Novak, Shamia Farhana Shoma, Natália O. Leiner, Diego Astúa, Graeme Coulson, Diana O. Fisher, Natasha D Harrison, Julian Nicholas Garcia Willmer, Giulia R. Silva, Gabriel Cupolillo, Marcus Vinícius Vieira, Emerson Monteiro Vieira, Thomas Püttker, Marcos de Souza Lima Figueiredo, Christopher R. Dickman

Introduction

Marsupials represent the only extant group of Metatheria, constituting one of the three major lineages of living mammals, alongside monotremes and eutherians (placental mammals). Currently, there are 396 extant species of marsupials, distributed in the Americas and Australasia (Abreu et al. 2024; Astúa et al. 2023; AMTC 2024; Baker et al. 2023). Extant marsupials encompass many commonly-known and iconic species, including opossums, kangaroos, koala, possums, wombats, Tasmanian devils, and bandicoots. This diverse group of mammals includes species with a broad spectrum of adaptive strategies. They thrive in the driest deserts and in the wettest tropical forests, living in terrestrial, arboreal, subterranean, and aquatic environments; some are highly social while others are solitary; many are omnivorous, while others are carnivorous or herbivorous (Cáceres & Dickman 2023). However, marsupials differ from other mammals by their mode of reproduction and reproductive anatomy (Tyndale-Biscoe 2005). Young are born at an early stage of development, after a relatively short gestation period (Hayssen et al. 1985). For marsupial species, most growth of young and the majority of energy allocated to reproduction occurs during lactation, which is 40% longer than in eutherians of comparable body mass (Hayssen et al. 1985; Sunquist & Eisenberg 1993). These fundamental differences in the timing and mode of maternal energy allocation to reproduction have major consequences for marsupial ecology and conservation.

Marsupials face numerous challenges posed by anthropogenic changes to their environment (Lindenmayer & Dickman 2023; Doherty et al. 2023; Vieira et al. 2023). Despite the wide diversity of adaptive strategies of marsupials, rapid environmental changes make it uncertain whether species will be able to cope with such changes and avoid population decline or extinction. Over 10% (18 species) of Australian marsupials have already become extinct in the

past two centuries, a rate significantly above the global mammal average of 1.4% (Schipper et al. 2008). Alarming declines and extinctions of Australian marsupials have continued unabated since the mid nineteenth century and approximately 40% of Australian marsupials are currently classified as threatened (i.e. Vulnerable, Endangered or Critically Endangered) in the IUCN Red List (Woinarski & Fisher 2023). No marsupial species from New Guinea or Wallacea is recognized to have become extinct in the last 200 years, but two are currently flagged as "Possibly Extinct" (*Phalanger matanim* and *Dendrolagus mayri*; Woinarski & Fisher 2023). Although fewer in number, American marsupials are similarly threatened, with two species assigned as Critically Endangered (*Marmosops handleyi* and *Monodelphis unistriata*; IUCN 2025), while several others have not yet been assessed (Martin & Carmignotto 2024).

Compiling comprehensive databases is essential to enable identification of large-scale patterns, conduct robust comparative analyses, and support evidence-based conservation decisions (Strier et al. 2010; Runting et al. 2020). Despite their ecological and evolutionary importance, much of the data on the life history and ecology of marsupials are scattered across multiple sources (e.g. de Magalhães & Costa 2009; Jones et al. 2009; Wilman et al. 2014) or underrepresented in databases and comparative studies on mammals (e.g. Promislow & Harvey 1990; Heppell et al. 2000; Oli & Dobson 2003; Healy et al. 2019). Centralizing information on the ecology and life history of marsupials in a unified database represents a crucial step in promoting a deeper understanding of marsupial adaptations, and facilitating the inclusion of this group in global analyses of mammals, vertebrates, and the animal kingdom as a whole, and thereby contributing significantly to improving our understanding of this taxonomic group and subsequent ability to guide conservation efforts.

Here, we provide the most comprehensive database on the ecology and life history of marsupials developed by our working group - The Marsupial Database Initiative. The Marsupial Database stores a curated collection of data on 414 extant and recently extinct marsupial species from all seven modern orders (Dasyuromorphia, Didelphimorphia, Diprotodontia, Microbiotheria, Notoryctemorphia, Paucituberculata, Peramelemorphia) native to 34 countries in the Americas and Australasia. The database contains 11,054 records of 35 traits describing anatomical, physiological, phenological and reproductive characteristics, and information on species' ecology and current conservation status. Data were collected from 41 sources, comprising published databases and other relevant sources of information. A second file containing average values for continuous life history characteristics is also provided. By providing a centralized repository of data on marsupial ecology and life history, The Marsupial Database facilitates comparative analyses of ecological and evolutionary patterns within the group, and improves our understanding of marsupial trait diversity and the ecosystem functions they provide, in addition to filling in taxonomic gaps to aid conservation efforts of marsupials worldwide.

Class I. Data Set Descriptors

A. Data set identity: The Marsupial Database: A comprehensive dataset on the ecology and life history of American and Australasian marsupials

B. Data set identification code: trait_database.csv, trait_database_mean.csv

C. Data set description

1. Originators:

Mariana Silva Ferreira. Instituto de Biodiversidade e Sustentabilidade, Universidade Federal do Rio de Janeiro, Macaé, Rio de Janeiro, Brasil and Programa de Pós-Graduação em Ecologia, Departamento de Ecologia, Instituto de Biologia, Universidade Federal do Rio de Janeiro, Rio de Janeiro, Rio de Janeiro, Brasil.

Natália Oliveira Leiner. Instituto de Biologia, Universidade Federal de Uberlândia, Uberlândia, Minas Gerais, Brasil.

Marcus Vinícius Vieira. Instituto de Biologia, Departamento de Ecologia, Universidade Federal do Rio de Janeiro, Rio de Janeiro, Rio de Janeiro, Brasil.

Emerson Monteiro Vieira. Instituto de Biologia, Universidade de Brasília, Brasília, Distrito Federal, Brasil.

Diana O. Fisher. School of the Environment, University of Queensland, Brisbane, Queensland, Australia.

Christopher R. Dickman. School of Life and Environmental Sciences, The University of Sydney, Sydney, New South Whales, Australia.

2. Abstract:

Marsupials are an important but typically neglected group of mammals that have been overlooked in many comparative analyses of vertebrate ecology and life-history evolution. In order to address this knowledge bias, we have developed The Marsupial Database. The Marsupial Database contains traits for a phylogenetically diverse set of 414 extant and recently extinct (last 200 years) species from all seven modern marsupial orders (Dasyuromorphia, Didelphimorphia, Diprotodontia, Microbiotheria, Notoryctemorphia, Paucituberculata, Peramelemorphia) native to 34 countries in the Americas and Australasia. The database comprises 11,054 records of 35 traits describing

anatomical, physiological, phenological and reproductive characteristics, as well as information on species' ecology and current conservation status. Data were collected from 41 sources, comprising published databases and other relevant sources of information. By providing a centralized repository of marsupial ecological and life history traits, The Marsupial Database facilitates analyses of ecological and evolutionary patterns within the group, and also encourages inclusion of marsupials in comparative studies of mammals, vertebrates and the entire animal kingdom. The Marsupial Database is free from copyright or proprietary restrictions. Please cite this data paper when using the data in publications or scientific presentations.

D. Key words/phrases: body mass; comparative analyses; diet; macroecology; mammal; phenology; physiology; population density; reproduction; survival; trait; vertebrate.

Class II. Research origin descriptors

A. Overall project description:

1. **Identity:** Information on the ecology and life history of American and Australasian marsupials gathered from published databases, books, and papers with data compilation.

2. **Originators:**

Mariana Silva Ferreira. Instituto de Biodiversidade e Sustentabilidade, Universidade Federal do Rio de Janeiro, Macaé, Rio de Janeiro, Brasil and Programa de Pós-Graduação em Ecologia, Departamento de Ecologia, Instituto de Biologia, Universidade Federal do Rio de Janeiro, Rio de Janeiro, Rio de Janeiro, Brasil.

Natália Oliveira Leiner. Instituto de Biologia, Universidade Federal de Uberlândia, Uberlândia, Minas Gerais, Brasil.

Marcus Vinícius Vieira. Instituto de Biologia, Departamento de Ecologia, Universidade Federal do Rio de Janeiro, Rio de Janeiro, Rio de Janeiro, Brasil.

Emerson Monteiro Vieira. Instituto de Biologia, Universidade de Brasília, Brasília, Distrito Federal, Brasil.

Diana O. Fisher. School of the Environment, University of Queensland, Brisbane, Queensland, Australia.

Christopher R. Dickman. School of Life and Environmental Sciences, The University of Sydney, Sydney, New South Whales, Australia.

3. **Period of study:** Not applicable.

4. **Objectives:** To build a comprehensive database on the ecology and life history of American and Australasian marsupials. Our database is intended to serve as a resource for comparative studies and macroecological analyses.

5. **Abstract:** Same as above.

6. **Sources of funding:** The compilation of this dataset was supported by the Australian Alumni Grants Round 2023-2024 of the Australian Embassy in Brazil. MSF was supported by the Long-Term Ecological Research program (PELD, site MCF) of the National Council for Scientific and Technological Development of Brazil (Conselho Nacional de Desenvolvimento Científico e Tecnológico - CNPq). MVV was supported by grants from CNPq and Carlos Chagas Foundation for Research Support of the Rio de Janeiro State (Fundação Carlos Chagas de Amparo à Pesquisa do Estado do Rio de Janeiro - FAPERJ). DA was supported by grants from CNPq (307457/2025-7), the

Coordination for the Improvement of Higher Education Personnel (Coordenação de Aperfeiçoamento de Pessoal de Nível Superior - CAPES) and Foundation for the Support of Science and Technology of Pernambuco (Fundação de Amparo à Ciência e Tecnologia de Pernambuco - FACEPE). NL was supported by the Long-Term Ecological Research program (PELD, site TSMG) of CNPq, and Foundation for Research Support of Minas Gerais State (Fundação de Amparo à Pesquisa do Estado de Minas Gerais - FAPEMIG). JNGW was supported by CAPES.

B. Specific subproject description

1. Site description: Global trait data compilation for extant and recently extinct mammals from published sources.

2. Experimental or sampling design: Not applicable.

3. Research methods:

a. Taxonomy and systematics: Taxonomy and nomenclature were based on the updated checklist of American and Australasian marsupials found in Astúa et al. (2023) and Baker et al. (2023) respectively, the updated list of native species of Brazil conducted by the Taxonomy Committee of the Brazilian Society of Mammalogy (Abreu et al. 2024), and the current list provided by the Australasian Mammal Taxonomy Consortium (AMTC 2024). Nomenclatural mismatches due to taxonomic changes and misspellings were checked by experts in each marsupial order and were assigned to current taxonomic nomenclatures. Nomenclatural and taxonomic changes fell into two categories: genus and/or specific epithet change, and new species discovered. For the first, data were kept unchanged. For the second, no data were included when the species was recently described. When a new species resulting from a species split in taxonomy

occurred, we analyzed case-by-case and the data were linked to the appropriate species by analyzing the original source of data. For example, the genus *Dasycercus* was considered to comprise two species: *Dasycercus cristicauda* and *Dasycercus blythi*, with the previously named *Dasycercus hillieri* considered a junior synonym of *D. cristicauda*. Recently, Newman-Martin et al. (2023) confirmed the validity of *D. hillieri* and indicated that much of the modern *D. cristicauda* material should be reassigned to *D. hillieri*. Subspecies-specific data were not differentiated in this database. Data were unchanged and assigned to the species.

b. Data sources: A total of 41 data sources including published databases, books and papers with data compilation were used to build this database. Table 1 presents the databases used to build the Marsupial Database for each variable and Table 2 describes the format in which data are reported in the original sources (average values, individual measurements, or categorical data) and specifies whether sample size information is included.

Table 1. Data sources used to build the Marsupial Database. Identity - Unique variable name; Source - Data source.

Identity	Source
Adult body mass (g)	Amador & Gianini 2016; Ashwell 2008; Battistella et al. 2019; Buckley et al. 2018; Cássia-Silva & Sales 2019; Collett 2018; de Magalhaes & Costa 2009; Faurby et al. 2018; Fisher et al. 2001; Gonzalez-Voyer et al. 2016; Heldstab et al. 2018; Jackson 2003; Jones et al. 2009; McNab 2008; Moura et al. 2024; Myhrvold et al. 2015; Pacifici et al. 2013; Paglia et al. 2012; Soria et al. 2021; Todorov et al. 2021; Williams et al. 2010; Wilman et al. 2014; Wilson & Mittermeier 2015[1]
Adult female body	Ferreira et al. 2023; Fisher et al. 2001; Myhrvold et al. 2015;

mass (g)	Russell 1982; Russell 1984; Smith & Lee 1984; Weisbecker et al. 2013; Williams et al. 2010; Wilson & Mittermeier 2015[1]
Adult male body mass (g)	Jackson 2003; Jones et al. 2009; Myhrvold et al. 2015; Rose et al. 1997; Weisbecker et al. 2013; Williams et al. 2010; Wilson & Mittermeier 2015[1]
Gestation length (d)	Cássia-Silva & Sales 2019; Collett 2018; de Magalhães & Costa 2009; Fisher et al. 2001; Jackson 2003; Jones et al. 2009; Myhrvold et al. 2015; Soria et al. 2021; Wilson & Mittermeier 2015[1]
Number of neonates	Wilson & Mittermeier 2015[1]
Litter size	Battistella et al. 2019; Cássia-Silva & Sales 2019; Collett 2018; de Magalhães & Costa 2009; Eisenberg & Wilson 1981; Ferreira et al. 2023; Fisher et al. 2001; Gonzalez-Voyer et al. 2016; Jackson 2003; Jones et al. 2009; Myhrvold et al. 2015; Russell 1982; Soria et al. 2021; Todorov et al. 2021; Williams et al. 2010; Wilson & Mittermeier 2015[1]
Number of reproductive events (y)	Cássia-Silva & Sales 2019; Collett 2018; de Magalhães & Costa 2009; Jackson 2003; Jones et al. 2009; Myhrvold et al. 2015; Smith & Lee 1984; Soria et al. 2021; Williams et al. 2010; Wilson & Mittermeier 2015[1]
Generation length (d)	Soria et al. 2021; Pacifici et al. 2013
Age at weaning (d)	Cássia-Silva & Sales 2019; de Magalhães & Costa 2009; Fisher et al. 2001; Gonzalez-Voyer et al. 2016; Hood 2020; Jackson 2003; Jones et al. 2009; Myhrvold et al. 2015; Smith & Lee 1984; Soria et al. 2021; Todorov et al. 2021; Wilson & Mittermeier 2015[1]
Inter-birth interval (d)	de Magalhães & Costa 2009; Fisher et al. 2001; Jones et al. 2009; Myhrvold et al. 2015; Soria et al. 2021; Wilson & Mittermeier 2015[1]
Age at first reproduction (d)	de Magalhães & Costa 2009; Ferreira et al. 2023; Fisher et al. 2001; Hood 2020; Jackson 2003; Jones et al. 2009; Myhrvold et al. 2015; Pacifici et al. 2013; Soria et al. 2021; Williams et al. 2010; Wilson & Mittermeier 2015[1]
Age at last reproduction (d)	Ferreira et al. 2023; Wilson & Mittermeier 2015[1]
Maximum lifespan (y)	Carey & Judge 2000; Collett 2018; de Magalhães & Costa 2009; Fisher et al. 2001; Hood 2020; Jones et al. 2009; Myhrvold et al. 2015; Pacifici et al. 2013; Smith & Lee 1984; Soria et al. 2021; Tidiere et al. 2016; Williams et al. 2010; Wilson & Mittermeier 2015[1]

Mass at birth (g)	de Magalhães & Costa 2009; Fisher et al. 2001; Jones et al. 2009; Myhrvold et al. 2015; Russell 1984; Soria et al. 2021
Mass at pouch vacation (g)	Fisher et al. 2001; Russell 1982
Mass at weaning (g)	de Magalhães & Costa 2009; Fisher et al. 2001; Jones et al. 2009; Myhrvold et al. 2015; Russell 1982; Smith & Lee 1984; Soria et al. 2021
Mass at maturity (g)	Fisher et al. 2001; Myhrvold et al. 2015; Wilson & Mittermeier 2015[1]
Testicular mass (g)	Rose et al. 1997; Wilson & Mittermeier 2015[1]
Pouch-young survival (%)	Russell 1982
Juvenile survival (%)	Ferreira et al. 2023; Russell 1982; Wilson & Mittermeier 2015[1]
Adult survival (%)	Ferreira et al. 2023
Mean group size	Fisher et al. 2001; Jones et al. 2009; Qiu et al. 2022; Wilson & Mittermeier 2015[1]
Social organization	Qiu et al. 2022; Russell 1984; Todorov et al. 2021; Wilson & Mittermeier 2015[1]
Social system categorization	Jackson 2003; Qiu et al. 2022; Russell 1984; Smith & Lee 1984; Todorov et al. 2021; Wilson & Mittermeier 2015[1]
Mating system	Rose et al. 1997; Russell 1984; Smith & Lee 1984; Todorov et al. 2021; Wilson & Mittermeier 2015[1]
Basal metabolic rate (mLO^2/h)	Ashwell 2008; Buckley et al. 2018; de Magalhães & Costa 2009; Jones et al. 2009; McNab 2008
Field metabolic rate (mL CO_2/g/h)	Ashwell 2008; Todorov et al. 2021
Hibernation/torpor	Buckley et al. 2018; Heldstab et al. 2018; McNab 2008; Soria et al. 2021; Todorov et al. 2021; Turbill et al. 2011; Wilson & Mittermeier 2015[1]
Population density (ha-1)	Fisher et al. 2001; Gonzalez-Voyer et al. 2016; Jones et al. 2009; Santini et al. 2018; Soria et al. 2021; Todorov et al. 2021; Wilson & Mittermeier 2015[1]
Activity period	Ferreira 2023; Fisher et al. 2001; Jones et al. 2009; Russell 1984; Soria et al. 2021; Todorov et al. 2021; Williams et al. 2010; Wilman et al. 2014; Wilson & Mittermeier 2015[1]

Shelter type	Fisher et al. 2001; Todorov et al. 2021; Williams et al. 2010; Wilson & Mittermeier 2015[1]
Strata use	Fisher et al. 2001; Paglia et al. 2012; Soria et al. 2021; Williams et al. 2010; Wilman et al. 2014; Wilson & Mittermeier 2015[1]
Diet type	Fisher et al. 2001; Gainsbury et al. 2018; Kissling et al. 2014; Pineda-Munoz & Alroy 2014; Soria et al. 2021; Todorov et al. 2021; Williams et al. 2010; Wilman et al. 2014; Wilson & Mittermeier 2015[1]
Habitat	IUCN 2025[2]; Wilson & Mittermeier 2015[1]
IUCN red list categories	IUCN 2025[2]

[1] The Wilson & Mittermeier (2015) data come from data compiled by several authors in each chapter of the book.

[2] The IUCN (2025) data were obtained from each assessment conducted by multiple authors for each species.

Table 2. Summary of how trait data are presented in the original sources, including the type of data reported (average or raw measurements, or categorical data) and the availability of sample size information. For data sources with information on species' longevity, only maximum values were recorded (*). - = the information was not available.

Data source	Data reported in the original source	Sample size availability
Amador & Gianini 2016	average	no
Ashwell 2008	raw/average	yes
Battistella et al. 2019	average	yes
Buckley et al. 2018	raw/categorical	no
Carey & Judge 2000	raw*	no
Cássia-Silva & Sales 2019	average	no
Collett et al. 2018	average (when possible)	no
de Magalhaes & Costa 2009	raw*/average	no

Eisenberg & Wilson 1981	raw*/average	yes
Faurby et al. 2018	average (when possible)/categorical	no
Ferreira 2023	categorical	yes
Ferreira et al. 2023	average	yes
Fisher et al. 2001	raw/average	no
Gainsbury et al. 2018	categorical	no
Gonzalez-Voyer et al. 2016	raw/average	no
Heldstab et al. 2018	average/categorical	no
Hood 2020	-	no
IUCN 2025	categorical	no
Jackson 2003	raw/average	no
Jones et al. 2009	raw*/average/categorical	no
Kissling et al. 2014	categorical	no
McNab 2008	raw/categorical	no
Moura et al. 2024	raw/categorical	no
Myhrvold et al. 2015	raw*/categorical	no
Pacifici et al. 2013	raw*/average	no
Paglia et al. 2012	-	no
Pineda-Munoz & Alroy 2014	average	no
Qiu et al. 2022	categorical	no
Rose et al. 1997	average/categorical	yes
Russell 1982	raw	no
Russell 1984	raw/categorical	no
Santini et al. 2018	raw	no
Smith & Lee 1984	raw/average	no
Soria et al. 2021	average/categorical	no

Tidière et al. 2016	average	no
Todorov et al. 2021	raw/average/categorical	no
Turbill et al. 2011	raw/categorical	no
Weisbecker et al. 2013	average	no
Williams et al. 2010	raw/categorical	no
Wilman et al. 2014	average/categorical	no
Wilson & Mittermeier 2015	raw/average	no

c. Data compilation: We conducted a bibliographical search for trait databases and data included in other relevant publications in August 2024. We searched for relevant data combining keywords relevant to the target group (“marsupial*”, “mammal*”, “vertebrate*”), the type of source (“database*”, “dataset*”, “data”) and the target information (“trait*”, “life-history trait*”, “ecology”) in Web of Science and Google Scholar. We also included other sources discovered by the snowball principle, i.e. papers and databases cited in the selected sources, and indicated by our group of experts. We reviewed 41 of these data sources, resulting in 11,054 data points on the ecology and life history of marsupials.

Whenever possible, we collected three separate measurements or observations for each species. This approach was intended to capture variability within the species and get a more representative picture of the trait's value for that species. Some data sources presented both species’ average data and raw data (Table 2). Throughout the data collection, we opted to compute the mean value of the trait per species; raw data were only included when these were the only source of information. We also prioritized the most accurate data (i.e., included as many decimal places as were available) whenever

possible, and selected data from wild specimens rather than captive ones. We found that many data sources included information from the same databases and other similar sources. To avoid pseudoreplication, we checked all the original data sources and removed overlapping or conflicting data. We considered only species-specific reported data, i.e. direct observations. Imputed data, and mean values of congenerics and confamilials were not included in the database. For every species, the variable-specific source is referenced in a separate source dataset (trait_sources.csv).

d. Data transformation: Most traits were homogenous amongst sources and could be coalesced together with minimal (i.e. only changing measurement units) or no transformations. The following traits required minimal or more complex transformations:

- **Adult body mass, Adult male body mass and Adult female body mass, Mass at birth, Mass at pouch vacation, Mass at weaning, Mass at maturity and Testicular mass**: Data were converted from kilograms to grams. When range data were available, the maximum and minimum values were averaged.

- **Gestation length, Generation length, Age at weaning, Inter-birth interval, Age at first reproduction and Age at last reproduction:** Data were converted from years to days. When range data were available, the maximum and minimum values were averaged.

- **Generation length:** Some data on generation length originated from the Pacifici et al. (2013) database on Generation length for Mammals, which estimated generation length from information on species' age at first reproduction and

reproductive life span by applying the methodology described in the IUCN Red List Guidelines. The following equation was used: $GL = R_{span} * z + AFR$, where R_{span} (reproductive life span) is calculated as the difference between the age at last reproduction and the age at first reproduction (AFR). The authors also used information on the maximum known longevity in the absence of data on age at last reproduction, since age at last reproduction is generally related to longevity in the wild. For more details, see Pacifici et al. (2013).

We also estimated generation length using the equation above and the data available in the database. For these estimations, we prioritize the use of age at last reproduction over maximum lifespan. When more than one value was available for age at first reproduction and age at last reproduction (or maximum lifespan), we used the average. These estimated values were identified separately in the reference column (Generation_length_ref) in the database as: Estimated_Pacifici_et_al._2013_ age-at-last-reproduction and Estimated_Pacifici_et_al._2013_maximum-lifespan.

- **Number of reproductive events**: Data were converted from events per day to events per year. When range data were available, the maximum and minimum values were averaged.
- **Maximum lifespan**: Data were converted from years to days.
- **Social organization categories**: In the absence of specific data on social organization, information presented in Todorov et al. (2021) was considered and species were classified as solitary or social.

- **Basal metabolic rate:** Data in Watts and kilojoules per hour were converted to mL O_2 h^{-1}. Values reported in Watts were converted assuming a factor of 179.1 mL O_2 h^{-1} W^{-1} and values reported in kilojoules per hour were converted assuming a factor of 49.75 mL O_2 h^{-1} kJ^{-1} (Buckley et al. 2018; Schmidt-Nielsen, 1997).

- **Field metabolic rate**: Data in kilojoules per day were converted to ml CO_2 g^{-1} h^{-1} assuming a factor of 47.4 ml CO_2 and dividing by the mean body mass of each species in the database (Schmidt-Nielsen, 1997).

- **Population density:** Data were converted from individuals/kilometer to individuals/hectare.

3. Project personnel:

Mariana Silva Ferreira. Instituto de Biodiversidade e Sustentabilidade, Universidade Federal do Rio de Janeiro, Macaé, Rio de Janeiro, Brasil and Programa de Pós-Graduação em Ecologia, Departamento de Ecologia, Instituto de Biologia, Universidade Federal do Rio de Janeiro, Rio de Janeiro, Rio de Janeiro, Brasil.

Rodrigo C. Rossi. Instituto de Biologia, Universidade Federal de Uberlândia, Uberlândia, Minas Gerais, Brasil.

Stephane Batista. Queensland University of Technology, Brisbane, Queensland, Australia.

Kathleen Sena. Instituto de Biodiversidade e Sustentabilidade, Universidade Federal do Rio de Janeiro, Macaé, Rio de Janeiro, Brasil.

Kevin R. Bairos-Novak. School of the Environment, University of Queensland, Brisbane, Queensland, Australia.

Shamia Farhana Shoma. School of the Environment, University of Queensland, Brisbane, Queensland, Australia.

Natália O. Leiner. Instituto de Biologia, Universidade Federal de Uberlândia, Uberlândia, Minas Gerais, Brasil.

Diego Astúa. Departamento de Zoologia, Universidade Federal de Pernambuco, Recife, Pernambuco, Brasil.

Graeme Coulson. School of BioSciences, University of Melbourne, Melbourne, Victoria, Australia.

Diana O. Fisher. School of the Environment, University of Queensland, Brisbane, Queensland, Australia.

Natasha D Harrison. School of Biological Sciences, University of Western Australia, Perth, Western Australia, Australia.

Julian Nicholas Garcia Willmer. School of Geography Earth and Environmental Sciences, University of Birmingham, Birmingham, United Kingdom.

Giulia R. Silva. Instituto de Biologia, Universidade de Brasília, Brasília, Distrito Federal, Brasil.

Gabriel Cupolillo. Programa de Pós-Graduação em Biodiversidade e Biologia Evolutiva, Instituto de Biologia, Universidade Federal do Rio de Janeiro, Rio de Janeiro, Rio de Janeiro, Brasil.

Marcus Vinícius Vieira. Instituto de Biologia, Departamento de Ecologia, Universidade Federal do Rio de Janeiro, Rio de Janeiro, Rio de Janeiro, Brasil.

Emerson Monteiro Vieira. Instituto de Biologia, Universidade de Brasília, Brasília, Distrito Federal, Brasil.

Thomas Püttker. Departamento de Ciências Ambientais, Instituto de Ciências Ambientais, Químicas e Farmacêuticas, Universidade Federal de São Paulo, Diadema, Brasil.

Marcos de Souza Lima Figueiredo. Instituto de Biologia, Departamento de Ecologia, Universidade Federal do Rio de Janeiro, Rio de Janeiro, Rio de Janeiro, Brasil; Departamento de Ecologia e Recursos Marinhos, Universidade Federal do Estado do Rio de Janeiro, Rio de Janeiro, Rio de Janeiro, Brasil.

Christopher R. Dickman. School of Life and Environmental Sciences, The University of Sydney, Sydney, New South Whales, Australia

Class III. Data set status and accessibility

A. Status

1. **Latest update: Date of last modification of data set:** December 2025

2. **Latest archive date: Date of last data set archival:** Not applicable.

3. **Metadata status: Date of last metadata update and current status:** Last update on December 9, 2025, version submitted.

4. **Data verification:** Data were checked and inconsistent values such as typographical errors, incorrectly spelled scientific names and trait measures that did not make sense biologically (cross-checked with the original descriptions) were either manually corrected (when possible) or removed from the database. Data verification was conducted by marsupial specialists as following: Natália Oliveira Leiner and Diego Astúa (Order Didelphimorphia), Natasha Harrison (Order Peramelemorphia), Christopher R. Dickman (Order Dasyuromorphia), Diana O. Fisher (Order Diprotodontia; possums and gliders) and Graeme Coulson (Order Diprotodontia; macropods). The taxonomic status of the species was verified by the expert authors. In the bibliographic records, taxonomic updates were made based on the most recent literature (see Research methods 2a).

B. Accessibility

1. **Storage location and medium:** Data and metadata are available as Supporting Information in Data S1. The dataset is also available in Figshare at https://doi.org/10.6084/m9.figshare.29626664

2. **Contact persons:** For general inquiries about the dataset, contact: Mariana Silva Ferreira. Federal University of Rio de Janeiro (UFRJ), Institute of Biodiversity and Sustainability, Macaé – RJ, Brazil. Email: msferreira84@gmail.com

3. **Copyright restrictions:** The Marsupial Database is free from copyright or proprietary restrictions. Please cite this data paper when using the data in publications or scientific presentations.

4. **Proprietary restrictions:**

a. **Release date:** Not applicable.

b. **Citations:**

Ferreira, M. S. et al. 2026. "The Marsupial Database: A comprehensive dataset on the ecology and life history of American and Australasian marsupials." *Ecology*.

Ferreira, M. S. et al. 2026. "The Marsupial Database: A comprehensive dataset on the ecology and life history of American and Australasian marsupials." *Figshare*. https://doi.org/10.6084/m9.figshare.29626664

c. **Disclaimer(s):** Please cite this data paper and all relevant underlying data sources (found in trait_database.csv) when the data are used in publications or scientific presentations. We also request that researchers inform us of how they are using the data.

5. **Costs:** None.

Class IV. Data structural descriptors

A. Data set file

1. **Identity:**

1. trait_database.csv
2. trait_database_mean.csv

2. Size:

1. 93,225 records (including header) and 75 fields. Total file size is 548 KB.
2. 12,035 records (including header) and 29 fields. Total file size is 69 KB.

3. Format and storage mode: The dataset is downloadable as a single zipped archive, Data_S1.zip (54 KB), which contains two data files stored as semicolon-separated values (.csv).

4. Header information: The first rows of all files contain variable names (see Class IV Section B, Table 3).

5. Alphanumeric attributes: Mixed.

6. Special characters/fields: Missing fields are coded as NA.

7. Authentication procedures: Expert validation and independent double-checking by researchers were conducted to ensure the accuracy of the database.

B. Variable information

1. Variable identity: See Table 3.

2. Variable definition: See Table 3.

3. Units of measurement: See Table 3.

Table 3. Variable information of trait_database.csv and trait_sources.csv. Identity - Unique variable name; Definition - precise definition of variables in the database; Data type - type of value that can be stored in a variable or data field; Values - values, range of values or number of categories; Completeness - total percentage of data fields filled in the database (percentage per species).

Identity:	**order**
Definition:	Order to which the species belongs.
Data type:	Character
Values:	7 order names

Completeness: 100% (100%)

family

Definition: Family to which the species belongs.

Data type: Character

Values: 22 family names

Completeness: 100% (100%)

genus

Definition: Genus to which the species belongs.

Data type: Character

Values: 91 genus names

Completeness: 100% (100%)

species

Definition: Specific epithet.

Data type: Character

Values: 382 specific epithet

Completeness: 100% (100%)

genus_species

Definition: Scientific name of a species.

Data type: Character

Values: 414 species name

Completeness: 100% (100%)

adult_body_mass_g

Definition: The mean body mass of adult individuals in grams (g).

Data type: Numeric (float)

Values: Estimates range from 4.25 g to 49,500.00 g

Completeness: 71.50% (83.33%)

adult_female_body_mass_g

Definition: The mean body mass of adult females in grams (g).

Data type: Numeric (float)

Values: Estimates range from 4.20 g to 31,900.00 g

Completeness: 36.63% (56.28%)

adult_male_body_mass_g

Definition: The mean body mass of adult males in grams (g).

Data type: Numeric (float)

Values: Estimates range from 4.20 g to 57,000.00 g

Completeness: 34.70% (56.28%)

gestation_length_d

Definition: The duration of fetal growth in days (d).

Data type: Numeric (float)

Values: Estimates range from 11 days to 63 days.

Completeness: 29.07% (41.06%)

number_of_neonates

Definition: Total number of offspring born per litter per female(s), counted immediately after birth.

Data type: Numeric (integer)

Values: Estimates range from 5 to 30 individuals.

Completeness: 1.29% (3.14%)

litter_size

Definition: Number of offspring born per litter per female(s), counted after birth, and attached to the teats.

Data type: Numeric (float)

Values: Estimates range from 1 to 16 individuals.

Completeness: 57.81% (67.15%)

number_of_reproductive_events_y

Definition: The average number of reproductive events/bouts per female per year known for that population.

Data type: Numeric (float)

Values: Estimates range from 0.5 to 4 events per year.

Completeness: 33.66% (47.10%)

generation_length_d

Definition: The average age of parents of the current cohort (i.e. newborn individuals in the population), reflecting the turnover rate of breeding individuals in a population in days (d).

Data type: Numeric (float)

Values: Estimates range from 297.66 days to 3,650.00 days.

Completeness: 13.52% (40.57%)

age_at_weaning_d

Definition: The age when primary nutritional dependency on the mother ends and independent foraging begins to make a major contribution to the offspring's energy requirements in days (d).

Data type: Numeric (float)

Values: Estimates range from 12 days to 900 days.

Completeness: 34.86% (44.93%)

inter-birth_interval_d

Definition: The length of time between successive births by the same female(s) after a successful litter in days (d).

Data type: Numeric (float)

Values: Estimates range from 52.50 days to 851.00 days.

Completeness: 29.87% (37.68%)

age_at_first_reproduction_d

Definition: The age of first successful copulation leading to the birth of young in days (d).
This variable does not include gestation and is only recorded for females.

Data type: Numeric (float)

Values: Estimates range from 49 days to 1,440.00 days.

Completeness: 32.69% (42.03%)

age_at_last_reproduction_d

Definition: The age when no successful copulation occurred from that period onwards in days (d).
This variable is only recorded for females.

Data type: Numeric (float)

Values: Estimates range from 474.50 days to 5,110.00 days.

Completeness: 4.51% (9.18%)

maximum_lifespan_y

Definition: The oldest age recorded for a species in years (y). Maximum lifespan represents a single data point, not an average. Estimates are recorded for wild and captive populations, males and females.

Data type: Numeric (float)

Values: Estimates range from 1 year to 30.40 years.

Completeness: 34.70% (51.21%)

mass_at_birth_g

Definition: Mass of individual young immediately after birth or up to an age of seven
days after birth in grams (g).

Data type: Numeric (float)

Values: Estimates range from 0.0049 g to 1 g

Completeness: 12.96% (17.87%)

mass_at_pouch_vacation_g

Definition: Mass of individual young after the permanent exit from the pouch or after young permanently left in a nest in grams (g).
Estimates are recorded for males and females.

Data type: Numeric (float)

Values: Estimates range from 0.5 g to 5,700.00 g

Completeness: 8.45% (16.18%)

mass_at_weaning_g

Definition: Mass of individual young after weaning in grams (g). Estimates are recorded for males and females.

Data type: Numeric (float)

Values: Estimates range from 2 g to 12,000.00 g

Completeness: 18.84% (27.78%)

mass_at_maturity_g

Definition: Mass of individuals of first successful copulation leading to the birth of young in grams (g). Estimates are recorded for males and females.

Data type: Numeric (float)

Values: Estimates range from 5 g to 22,000.00 g

Completeness: 4.11% (11.11%)

testicular_mass_g

Definition: The testis mass of an adult male in grams (g).

Data type: Numeric (float)

Values: Estimates range from 0.05 g to 54.57 g

Completeness: 3.38% (9.66%)

pouch-young_survival_%

Definition: Percentage of offspring that survived the initial phase of development (from birth to weaning).
Estimates are recorded for females.

Data type: Numeric (float)

Values: Estimates range from 38% to 100%

Completeness: 0.72% (2.17%)

juvenile_survival_%

Definition: Percentage of young (not yet capable of reproduction) that survived the post-weaning phase.
Estimates are recorded for females.

Data type: Numeric (float)

Values: Estimates range from 10% to 96%

Completeness: 4.11% (7.97%)

adult_survival_%

Definition: Percentage of surviving adults after first reproduction.
Estimates are recorded for females.

Data type: Numeric (float)

Values: Estimates range from 50% to 100%

Completeness: 3.62% (7.00%)

mean_group_size

Definition: Number of individuals in a group that spend most of their daily time together.

Data type: Numeric (float)

Values: Estimates range from 1 to 25 individuals

Completeness: 19.24% (38.41%)

social_organization

Definition: The social patterns within a population, i.e., the set of individuals that interact with each other on different levels.

Data type: Ordinal

Values: Species were classified in eight categories following Russell (1984) and Qiu et al. (2022):

- S (solitary) – species in which individuals typically live and forage alone, interacting with conspecifics mainly during the breeding season.
- MF (pair-living) – two individuals, typically a male and a female, that live or regularly associate in stable or semi-stable groups, showing consistent social interactions beyond the breeding season,

and four forms of group-living

- MFF – single male multiple female group
- FMM – single female multiple male group
- sex-specific group: group of only males or only females
- MMFF – multi-male multi-female group
- G – group-living with unknown composition
  Combinations of categories – when more than one social organization was recorded (e.g., MF_MFF)

Completeness: 24.40% (42.03%)

social_organization_categories

Definition: Same as above.

Data type: Binary

Values: Species were classified in three categories:

- solitary – same as above

- social – species that live or regularly associate in stable or semi-stable groups, showing consistent social interactions beyond the breeding season.
- solitary_social – both social organization categories were recorded

Completeness: 26.89% (42.51%)

mating_system

Definition: The general pattern by which males and females mate.

Data type: Ordinal

Values: Species were classified in five categories:

- polygynous – one male mating with multiple females or harem
- polyandrous – one female mates with multiple males
- promiscuous – females mate with multiple males, and males mate with multiple females
- monogamous – male and one female are paired for at least one breeding season
- complex – polygamous/monogamous

Completeness: 12.00% (24.88%)

basal_metabolic_rate_mlO2/h

Definition: The rate of energy expenditure per unit time at rest, measured at thermoneutrality (i.e., experiencing neither heat nor cold stress) in milliliters of oxygen per hour (mlO_2/h).

Data type: Numeric (float)

Values: Estimates range from 10.39 mlO_2/h to 5,921.05 mlO_2/h

Completeness: 15.22% (18.36%)

field_metabolic_rate_mlCO2/g/h

Definition: The total rate of
energy expenditure by a free-living animal in a natural environment in milliliters of carbon dioxide per gram per hour (mlCO_2/g/h).

Data type: Numeric (float)

Values: Estimates range from 0.11 mlCO_2/g/h to 8.46 mlCO_2/g/h

Completeness: 4.91% (9.18%)

hibernation_torpor

Definition: Physiologically controlled reduction of metabolic rate and body temperature experienced by small endotherms when facing adverse periods, such as cold temperatures, food shortages and droughts. Torpor lasts less than 24 hours, while hibernation is defined by bouts of inactivity lasting from some days to several weeks (Ruf and Geiser 2015). We grouped together both types of adaptations, considering them as an indicator of avoidance of adverse environmental conditions as in Soria et al. (2021).

Data type: Ordinal

Values: Species were classified in four categories:

- hibernation – a prolonged seasonal state of reduced metabolism and body temperature
- torpor – a short-term reduction in metabolism and body temperature to conserve energy
- hibernation_torpor – physiological adaptation not specified
- no – no record of hibernation/torpor

Completeness: 36.07% (60.63%)

population_density_ha-1

Definition: Number of individuals per hectare (ind/ha).

Data type: Numeric (float)

Values: Estimates range from 0.02 ind/ha to 297 ind/ha.

Completeness: 20.21% (30.92%)

activity_period

Definition: Time of the day in which a species carries out most of its activities.

Data type: Ordinal

Values: Species were classified in five categories following Bennie et al. (2014):

- diurnal – majority of activity occurs during the day
- nocturnal – majority of activity occurs during the night
- crepuscular-nocturnal – majority of activity occurs during twilight (periods of dawn and dusk) or not fully nocturnal
- crepuscular-diurnal – majority of activity occurs during twilight – periods of dawn and dusk) or not fully diurnal
- cathemeral – activity during the day and night

Completeness: 58.62% (78.26%)

shelter_type

Definition: Type of shelter or refuge species are known to use.

Data type: Ordinal

Values: Species were classified into three categories following Fisher et al. (2001):

- protected – burrow or constructed nest in tree hollow
- intermediate – tree canopy, hollow log, under rock, nest on ground or in soil crack
- open – under shrubs, in grass or shade of tree

Completeness: 35.67% (52.90%)

strat_use

Definition: Primary type of strata of habitat used by a species.

Data type: Ordinal

Values: Species were classified in six categories following Williams et al. (2010):

- arboreal – primarily live or move in trees
- terrestrial – primarily live or move on the ground
- scansorial – use both trees and the ground for locomotion or foraging
- fossorial – primarily live underground (species adapted for digging)
- freshwater-terrestrial – use both freshwater habitats and adjacent terrestrial areas
- volant – capable of active flight

Completeness: 41.63% (82.13%)

diet_type

Definition: Broad dietary classification (*dietary guild*) of each species, based on the main proportion of their diet.

Data type: Ordinal

Values: Species were classified in four broad categories following Fisher et al. 2001:

- 1 – >50% grass or browse: *herbivorous*
- 2 – seeds, grass, roots, leaves, fruit, and invertebrates: *omnivorous*
- 3 – nectar or fruit with invertebrates: *omnivorous*
- 4 – >50% invertebrates or vertebrates: *carnivorous*

Completeness: 62.40% (79.71%)

habitat_type

Definition: The major habitat/s in which a species occurs.

Data type: Ordinal

Values: Species were classified in 10 broad categories following IUCN classification (2025):

- forest
- savanna
- shrubland
- grassland
- wetlands
- rocky areas
- caves and subterranean habitats
- desert
- artificial
- combinations of categories were also possible when the species was found in more than one habitat type. Categories were separated by an underscore.

Completeness: 28.66% (83.57%)

IUCN_red_list_categories

Definition: The IUCN Red List categories of species at high risk of global extinction.

Data type: Ordinal

Values: Species were classified in nine categories using IUCN classification obtained in February 2025:

- NE – Not Evaluated
- DD – Data Deficient
- LC – Least Concern
- NT – Near Threatened
- VU – Vulnerable
- EN – Endangered
- CR – Critically Endangered
- EW – Extinct in the Wild
- EX – Extinct

Completeness: 100% (83.09%)

4. Data type

a. Storage type: See Table 3 in section Class IV. B. Data type.

b. List and definition of variable codes: See Table 3 in section Class IV. B. Values.

c. Range for numeric values: See Table 3 in section Class IV. B. Values.

d. Missing value codes: Missing information was classified as "NA".

e. Precision: From 2 to 6, according to each variable.

Class V. Supplemental descriptors

A. Data summary:

This database evaluates a total of 414 marsupial species, comprising 128 species of the order Didelphimorphia, 3 of Microbiotheria, 7 of Paucituberculata, 2 of Notoryctemorphia, 31 of Peramelemorphia, 87 of Dasyuromorphia, and 156 of Diprotodontia. Among these, 18 species are known to be recently extinct (Peramelemorphia - 7 species; Dasyuromorphia - 4 species; Diprotodontia - 7 species). In total, 11,054 data records were compiled on 35 ecological and life-history traits from 41 different sources.

The order with the largest proportional number of records was Diprotodontia (33.61%), followed by Dasyuromorphia (30.07%; Figure 1). The three orders with the fewest records belonged to the American orders Didelphimorphia (15.71%), Microbiotheria (15.85%), and Paucituberculata (14.42%). The distribution of data among orders was not uniform (Figure 1). Data related to ecological traits were more common than data related to reproduction, anatomy, physiology, and survival. The latter data were the least abundant, regardless of the taxonomic group evaluated. However, this pattern is not general for all families (Figure 2). Families belonging to the order Diprotodontia, Phascolarctidae, Tarsipedidae, and Vombatidae, provided data for almost all traits in the database. In contrast, survival data are completely absent for some families present in the orders evaluated (Figure 2).

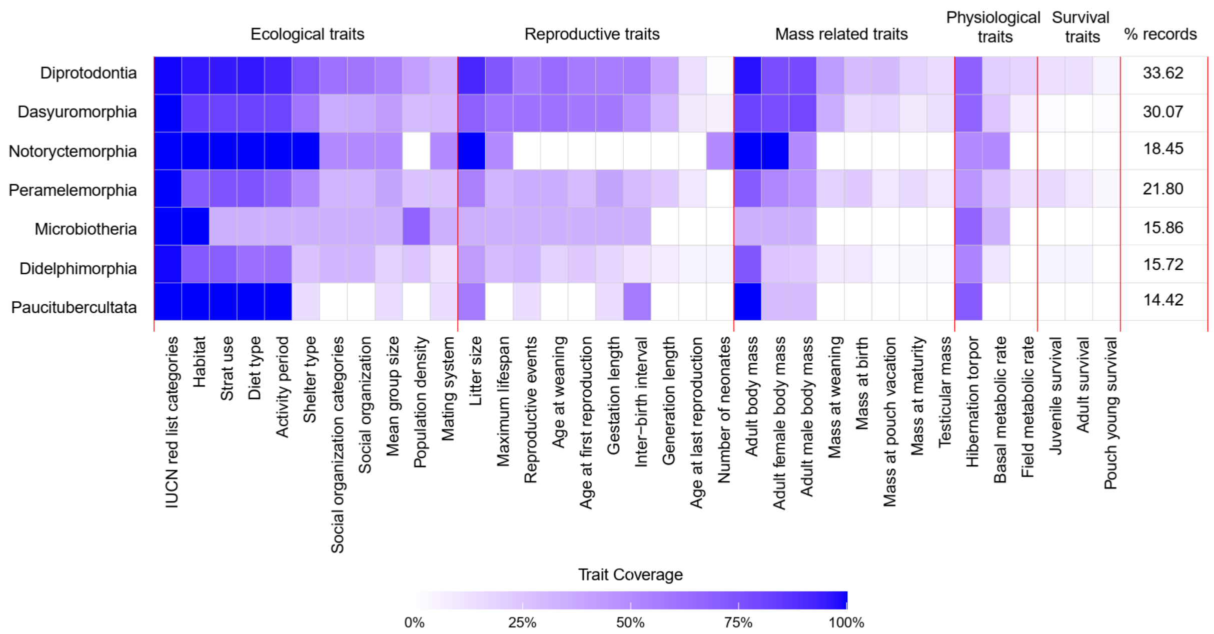

Figure 1. Ecological- and life-history trait coverage for seven marsupial orders present in The Marsupial Database. % records show how each order is represented in our database considering that the number of species varies across orders (For IUCN red list categories only one entry per species was considered). N = total number of records. Order Microbiotheria (N = 47), Paucituberculata (N = 104), Didelphimorphia (N = 2,069), Dasyuromorphia (N = 2,698), Notoryctemorphia (N = 38), Peramelemorphia (N = 696) and Diprotodontia (N = 5,402).

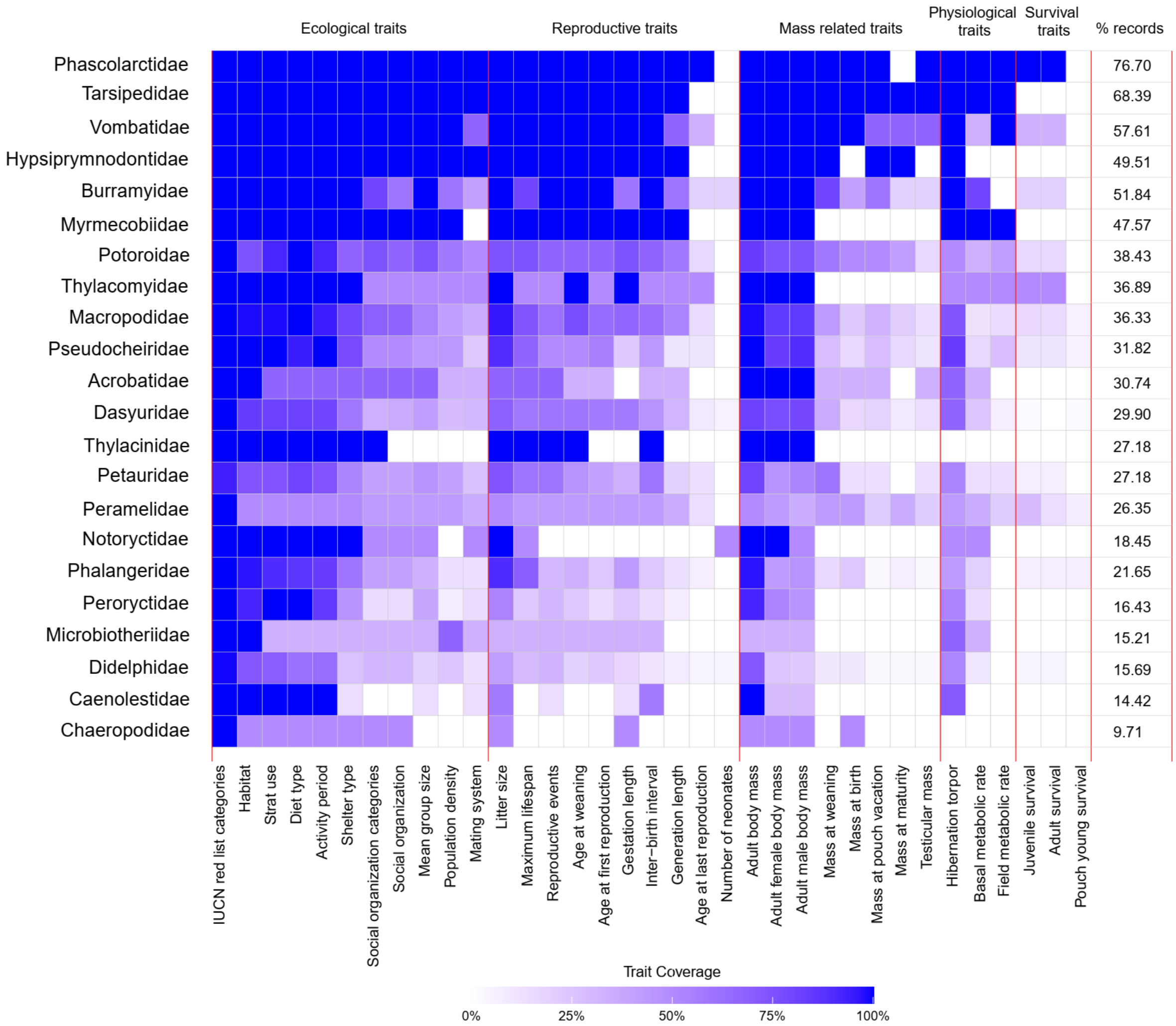

Figure 2. Ecological- and life-history trait coverage for 22 marsupial families present in The Marsupial Database.

B. Quality assurance/quality control procedures:

In addition to the quality control procedures carried out by the authors of the databases we used, all records were checked by the authors. Any errors identified in the original sources were removed.

To incorporate some of the natural variability present in the traits, we collected up to three independent data points per species whenever possible. This approach aimed to capture part of the

variation inherent to biological traits such as body mass, litter size and population density. We acknowledge, however, that this strategy is not sufficient to fully represent intraspecific variation, particularly for species with broad geographic distributions or those occurring across contrasting habitats. Nevertheless, having general ecological and life-history information available can support researchers in developing analyses of ecological and evolutionary patterns within the group, and may also encourage the inclusion of marsupials in comparative studies of mammals, vertebrates and the broader animal kingdom. For species- or site-specific studies, we recommend consulting more specialized data sources.

This database could be improved in the future by conducting an exhaustive literature search for each species, including extracting trait information stratified by geographic region. Such an effort would allow the incorporation of environmental, ecological, and spatial factors known to influence trait variability, thereby producing a more comprehensive and geographically explicit dataset. We consider this a valuable opportunity for future researchers working with marsupials or large-scale trait synthesis. In addition, as trait databases continue to grow in scope, the development of standardized reporting formats and clearer metadata requirements across primary studies would further enhance data quality and consistency, ultimately improving the reliability of future compilations.

C. Related material: N/A.

D. Computer programs and data-processing algorithms:

All data were initially compiled and organized in Excel spreadsheets, where preliminary checks for consistency and completeness were performed. Subsequent data processing, including data transformation, calculation of summary metrics, and harmonisation of variable formats, was

conducted in the R environment. All figures presented in the Data Paper were also generated in R using plotting packages.

References

Abreu, E. F., D. Casali, R. Costa-Araújo, G. S. T. Garbino, G. S. Libardi, D. Loretto, A. C. Loss, M. Marmontel, L. M. Moras, M. C. Nascimento, M. L. Oliveira, S. E. Pavan and F. P. Tirelli. 2024. "Lista de Mamíferos do Brasil (2024-1)." *Zenodo* [Data set]. https://doi.org/10.5281/zenodo.14536925

AMTC. 2024. "The AMTC Australian Mammal Species List," version 4.2. Australian Mammal Taxonomy Consortium. https://australianmammals.org.au/publications/amtc-species-list (Downloaded 01 August 2024)

Astúa, D., J. J. Cherem and P. Teta. 2023. "Taxonomic checklist of living American marsupials." In *American and Australasian Marsupials: An Evolutionary, Biogeographical, and Ecological Approach*, edited by N. C. Cáceres and C. R. Dickman, 115–162. Cham, Switzerland: Springer International Publishing.

Baker, A. M., M. D. Eldridge, D. O. Fisher, G. Frankham, K. Helgen, S. M. Jackson, … and L. S. Umbrello. 2023. "Taxonomy and diversity of living Australasian marsupials." In *American and Australasian Marsupials: An Evolutionary, Biogeographical, and Ecological Approach*, edited by N. C. Cáceres and C. R. Dickman, 163–247. Cham, Switzerland: Springer International Publishing.

Bennie, J. J., J. P. Duffy, R. Inger and K. J. Gaston. 2014. "Biogeography of time partitioning in mammals." *Proceedings of the National Academy of Sciences* 111(38): 13727–13732. https://doi.org/10.1073/pnas.1216063110

Bi, S., X. Zheng, X. Wang, N. E. Cignetti, S. Yang and J. R. Wible. 2018. "An Early Cretaceous eutherian and the placental–marsupial dichotomy." *Nature* 558(7710): 390–395. https://doi.org/10.1038/s41586-018-0210-3

Buckley, L. B., I. Khaliq, D. L. Swanson and C. Hof. 2018. "Does metabolism constrain bird and mammal ranges and predict shifts in response to climate change?" *Ecology and Evolution* 8(24): 12375–12385. https://doi.org/10.1002/ece3.4537

Cáceres, N. C. and C. R. Dickman. 2023. “American and Australasian marsupials: An Introduction.” In *American and Australasian Marsupials: An Evolutionary, Biogeographical, and Ecological Approach*, edited by N. C. Cáceres and C. R. Dickman, 3–20. Cham, Switzerland: Springer International Publishing.

de Magalhães, J. P. and J. Costa. 2009. “A database of vertebrate longevity records and their relation to other life-history traits.” *Journal of Evolutionary Biology* 22: 1770–1774. https://doi.org/10.1111/j.1420-9101.2009.01783.x

Doherty, T. S., W. L. Geary, V. Miritis and D. J. Watchorn. 2023. “Multiple threats affecting the marsupials of Australasia: Impacts and management.” In *American and Australasian Marsupials: An Evolutionary, Biogeographical, and Ecological Approach*, edited by N. C. Cáceres and C. R. Dickman, 1531–1554. Cham, Switzerland: Springer International Publishing.

Fisher, D. O., I. P. F. Owens and C. N. Johnson. 2001. “The ecological basis of life history variation in marsupials.” *Ecology* 82: 3531–3540. https://doi.org/10.2307/2680170

Hayssen, V., R. C. Lacy and P. J. Parker. 1985. “Metatherian reproduction: transitional or transcending?” *The American Naturalist* 126(5): 617–632. https://doi.org/10.1086/284443

Healy, K., T. H. Ezard, O. R. Jones, R. Salguero-Gómez and Y. M. Buckley. 2019. “Animal life history is shaped by the pace of life and the distribution of age-specific mortality and reproduction.” *Nature Ecology and Evolution* 3(8): 1217–1224. https://doi.org/10.1038/s41559-019-0938-7

Heppell, S. S., H. Caswell and L. B. Crowder. 2000. “Life histories and elasticity patterns: perturbation analysis for species with minimal demographic data.” *Ecology* 81(3): 654–665. https://doi.org/10.1890/0012-9658(2000)081[0654:LHAEPP]2.0.CO;2

IUCN. 2025. “The IUCN Red List of Threatened Species.” Gland, Switzerland: International Union for Conservation of Nature. https://www.iucnredlist.org

Jones, K. E., J. Bielby, M. Cardillo, S. A. Fritz, J. O’Dell, C. D. L. Orme, K. Safi, W. Sechrest, E. H. Boakes, C. Carbone, … et al. 2009. “PanTHERIA: a species level database of life history,

ecology, and geography of extant and recently extinct mammals." *Ecology* 90: 2648. https://doi.org/10.1890/08-1494.1

Lee, A. K. and A. Cockburn. 1985. *Evolutionary Ecology of Marsupials*. Cambridge, UK: Cambridge University Press.

Lindenmayer, D. and C. Dickman. 2022. "Impact of Habitat Loss and Fragmentation on Assemblages, Populations, and Individuals of Australasian Marsupials." In *American and Australasian Marsupials: An Evolutionary, Biogeographical, and Ecological Approach*, edited by N. C. Cáceres and C. R. Dickman, 1413–1444. Cham, Switzerland: Springer International Publishing.

Martin, G. M. and A. P. Carmignotto. 2025. "Taxonomic assessment, conservation status, and future perspectives for New World Marsupials." *Mammal Review* 55(1): e12366. https://doi.org/10.1111/mam.12366

Newman-Martin, J., K. J. Travouillon, N. Warburton, M. Barham and A. J. Blyth. 2023. "Taxonomic review of the genus *Dasycercus* (Dasyuromorphia: Dasyuridae) using modern and subfossil material; and the description of three new species." *Alcheringa: An Australasian Journal of Palaeontology* 47(4): 624–661. https://doi.org/10.1080/03115518.2023.2262083

Oli, M. K. and F. S. Dobson. 2003. "The relative importance of life-history variables to population growth rate in mammals: Cole's prediction revisited." *The American Naturalist* 161(3): 422–440. https://doi.org/10.1086/367591

Pacifici, M., L. Santini, M. Di Marco, D. Baisero, L. Francucci, G. G. Marasini, P. Visconti and C. Rondinini. 2013. "Generation length for mammals." *Nature Conservation* 5: 87–94.

Promislow, D. E. and P. H. Harvey. 1990. "Living fast and dying young: A comparative analysis of life-history variation among mammals." *Journal of Zoology* 220(3): 417–437. https://doi.org/10.1111/j.1469-7998.1990.tb04316.x

Qiu, J., C. A. Olivier, A. V. Jaeggi and C. Schradin. 2022. "The evolution of marsupial social organization." *Proceedings of the Royal Society B* 289: 20221589. https://doi.org/10.1098/rspb.2022.1589

Ruf, T. and F. Geiser. 2015. "Daily torpor and hibernation in birds and mammals." *Biological Reviews* 90(3): 891–926. https://doi.org/10.1111/brv.12137

Runting, R. K., S. Phinn, Z. Xie, O. Venter and J. E. Watson. 2020. "Opportunities for big data in conservation and sustainability." *Nature Communications* 11: 2003. https://doi.org/10.1038/s41467-020-15870-0

Schipper, J., J. S. Chanson, F. Chiozza, … et al. 2008. "The status of the World's land and marine mammals: Diversity, threat, and knowledge." *Science* 322: 225–230. https://doi.org/10.1126/science.11651

Schmidt-Nielsen, K. 1997. *Animal Physiology: Adaptation and Environment*. Cambridge, UK: Cambridge University Press.

Soria, C. D., M. Pacifici, M. Di Marco, S. M. Stephen and C. Rondinini. 2021. "COMBINE: a coalesced mammal database of intrinsic and extrinsic trait." *Ecology* 102: e03344. https://doi.org/10.1002/ecy.3344

Strier, K. B., J. Altmann, D. K. Brockman, A. M. Bronikowski, M. Cords, L. M. Fedigan, … and S. C. Alberts. 2010. "The primate life history database: A unique shared ecological data resource." *Methods in Ecology and Evolution* 1(2): 199–211. https://doi.org/10.1111/j.2041-210X.2010.00023.x

Sunquist, M. and J. Eisenberg. 1993. "Reproductive strategies of female *Didelphis*." *Bulletin of the Florida Museum of Natural History* 36(4): 109–140. https://doi.org/10.58782/flmnh.wqrp4162

Todorov, O. S., S. P. Blomberg, A. Goswami, K. Sears, P. Drhlík, J. Peters and V. Weisbecker. 2021. "Testing hypotheses of marsupial brain size variation using phylogenetic multiple imputations and a Bayesian comparative framework." *Proceedings of the Royal Society B* 288: 20210394. https://doi.org/10.1098/rspb.2021.0394

Tyndale-Biscoe, H. 2005. *Life of Marsupials*. CSIRO Publishing.

Vieira, M. V, Barros, C. S. and A. C. Delciellos. 2023. “Impact of Habitat Loss and Fragmentation in Assemblages, Populations, and Individuals of American Marsupials.” In *American and Australasian Marsupials: An Evolutionary, Biogeographical, and Ecological Approach*, edited by N. C. Cáceres and C. R. Dickman, 1319–1366. Cham, Switzerland: Springer International Publishing.

Wilman, H., J. Belmaker, J. Simpson, C. de la Rosa, M. M. Rivadeneira and W. Jetz. 2014. “EltonTraits 1.0: Species level foraging attributes of the world’s birds and mammals.” *Ecology* 95: 2027. https://doi.org/10.1890/13-1917.1

Woinarski, J. C. and D. O. Fisher. 2023. “Conservation biogeography of modern species of Australasian marsupials.” In *American and Australasian Marsupials: An Evolutionary, Biogeographical, and Ecological Approach*, edited by N. C. Cáceres and C. R. Dickman, 1367–1394. Cham, Switzerland: Springer International Publishing.

Data sources

Amador, L. I. and N. P. Giannini. 2020. “Evolution of diet in extant marsupials: emergent patterns from a broad phylogenetic perspective.” *Mammal Review* 51: 178–192. https://doi.org/10.1111/mam.12223

Ashwell, K. W. S. 2008. “Encephalization of Australian and New Guinean marsupials.” *Brain, Behavior and Evolution* 71: 181–199. https://doi.org/10.1159/000114406

Battistella, T., F. Cerezer, J. Bubadué, G. Melo, M. Graipel and N. Cáceres. 2019. “Litter size variation in didelphid marsupials: evidence of phylogenetic constraints and adaptation.” *Biological Journal of the Linnean Society* 126: 40–54. https://doi.org/10.1093/biolinnean/bly157

Buckley, L. B., I. Khaliq, D. L. Swanson and C. Hof. 2018. “Does metabolism constrain bird and mammal ranges and predict shifts in response to climate change?” *Ecology and Evolution* 8: 12375–12385. https://doi.org/10.1002/ece3.4537

Carey, J. R. and D. S. Judge. 2000. “Longevity records: life spans of mammals, birds, amphibians, reptiles, and fish.” Odense, DK: Odense University Press. https://www.demogr.mpg.de/longevityrecords/

Cassia-Silva, C. and L. P. Sales. 2019. “The imprints left by historical contingency on marsupials’ life-history traits.” *Journal of Zoology* 307: 149–158. https://doi.org/10.1111/jzo.12629

Collett, R. A., Baker, A. M., and D. O. Fisher 2018. “Prey productivity and predictability drive different axes of life-history variation in carnivorous marsupials.” *Proceedings of the Royal Society B*, 285(1890): 20181291. https://doi.org/10.1098/rspb.2018.1291

de Magalhães, J. P. and J. Costa. 2009. “A database of vertebrate longevity records and their relation to other life-history traits.” *Journal of Evolutionary Biology* 22: 1770–1774. https://doi.org/10.1111/j.1420-9101.2009.01783.x

Eisenberg, J. F. and D. E. Wilson. 1981. “Relative brain size and demographic strategies in didelphid marsupials.” *The American Naturalist* 118(1): 1–15.

Faurby, S. and J.-C. Svenning. 2015. “A species-level phylogeny of all extant and late Quaternary extinct mammals using a novel heuristic-hierarchical Bayesian approach.” *Molecular Phylogenetics and Evolution* 84: 14–26. https://doi.org/10.1016/j.ympev.2014.11.001

Ferreira, M. S. 2023. “Activity patterns of American marsupials.” In *American and Australasian Marsupials: An Evolutionary, Biogeographical, and Ecological Approach*, edited by N. C. Cáceres and C. R. Dickman, 1189–1219. Cham, Switzerland: Springer International Publishing.

Ferreira, M. S., C. R. Dickman, D. O. Fisher, M. de S. L. Figueiredo and M. V. Vieira. 2023. “Marsupial position on life-history continua and the potential contribution of life-history traits to population growth.” *Proceedings of the Royal Society B* 290: 20231316. https://doi.org/10.1098/rspb.2023.1316

Fisher, D. O., I. P. F. Owens and C. N. Johnson. 2001. “The ecological basis of life history variation in marsupials.” *Ecology* 82: 3531–3540. https://doi.org/10.2307/2680170

Gainsbury, A. M., O. J. S. Tallowin and S. Meiri. 2018. "An updated global data set for diet preferences in terrestrial mammals: testing the validity of extrapolation." *Mammal Review* 48: 160–167. https://doi.org/10.1111/mam.12119

Gonzalez-Voyer, A., M. González-Suárez, C. Vilà and E. Revilla. 2016. "Larger brain size indirectly increases vulnerability to extinction in mammals." *Evolution* 70: 1364–1375. https://doi.org/10.1111/evo.12943

Heldstab, S. A., K. Isler and C. P. van Schaik. 2018. "Hibernation constrains brain size evolution in mammals." *Journal of Evolutionary Biology* 31: 1582–1588. https://doi.org/10.1111/jeb.13353

Hood, G. A. 2020. "Semi-aquatic mammals: ecology and biology." Maryland, US: JHU Press.

IUCN. 2024. "The IUCN Red List of Threatened Species." International Union for Conservation of Nature. https://www.iucnredlist.org

Jackson, S. 2003. "Australian mammals: biology and captive management." Melbourne, AU: CSIRO Publishing.

Jones, K. E., J. Bielby, M. Cardillo, S. A. Fritz, J. O'Dell, C. D. L. Orme, K. Safi, W. Sechrest, E. H. Boakes, C. Carbone, … et al. 2009. "PanTHERIA: a species-level database of life history, ecology, and geography of extant and recently extinct mammals." *Ecology* 90: 2648. https://doi.org/10.1890/08-1494.1

Kissling, W. D., L. Dalby, C. Fløjgaard, J. Lenoir, B. Sandel, C. Sandom, K. Trøjelsgaard and J.-C. Svenning. 2014. "Establishing macroecological trait datasets: digitalization, extrapolation, and validation of diet preferences in terrestrial mammals worldwide." *Ecology and Evolution* 4: 2913–2930. https://doi.org/10.1002/ece3.1136

McNab, B. K. 2008. "An analysis of the factors that influence the level and scaling of mammalian BMR." *Comparative Biochemistry and Physiology* 151: 5–28. https://doi.org/10.1016/j.cbpa.2008.05.008

Moura, M. R., K. Ceron, J. J. M. Guedes, R. Chen-Zhao, Y. V. Sica, J. Hart, W. Dorman, J. M. Portmann, P. González-del-Pliego, A. Ranipeta, … et al. 2024. "A phylogeny-informed characterisation of global tetrapod traits addresses data gaps and biases." *PLOS Biology* 22: e3002658. https://doi.org/10.1371/journal.pbio.3002658

Myhrvold, N. P., E. Baldridge, B. Chan, D. Sivam, D. L. Freeman and S. K. M. Ernest. 2015. "An amniote life-history database to perform comparative analyses with birds, mammals, and reptiles." *Ecology* 96: 3109. https://doi.org/10.1890/15-0846R.1

Pacifici, M., L. Santini, M. Di Marco, D. Baisero, L. Francucci, G. G. Marasini, P. Visconti and C. Rondinini. 2013. "Generation length for mammals." *Nature Conservation* 5: 87–94. https://doi.org/10.3897/natureconservation.5.5734

Paglia, A. P., G. A. B. da Fonseca, A. B. Rylands, G. Herrmann, L. M. S. Aguiar, A. G. Chiarello, Y. L. R. Leite, L. P. Costa, S. Siciliano, M. C. M. Kierulff, … et al. 2012. "Lista anotada dos mamíferos do Brasil." 2nd edition. *Occasional Papers in Conservation Biology* 6: 1–76.

Pineda-Munoz, S. and J. Alroy. 2014. "Dietary characterization of terrestrial mammals." *Proceedings of the Royal Society B* 281: 20141173. https://doi.org/10.1098/rspb.2014.1173

Qiu, J., C. A. Olivier, A. V. Jaeggi and C. Schradin. 2022. "The evolution of marsupial social organization." *Proceedings of the Royal Society B* 289: 20221589. https://doi.org/10.1098/rspb.2022.1589

Rose, R. W., C. M. Nevison and A. F. Dixson. 1997. "Testes weight, body weight and mating systems in marsupials and monotremes." *Journal of Zoology* 243: 523–531. https://doi.org/10.1111/j.1469-7998.1997.tb02798.x

Russell, E. M. 1982. "Patterns of parental care and parental investment in marsupials." *Biological Reviews* 57: 423–486. https://doi.org/10.1111/j.1469-185X.1982.tb00704.x

Russell, E. M. 1984. "Social behaviour and social organization of marsupials." *Mammal Review* 14: 101–154. https://doi.org/10.1111/j.1365-2907.1984.tb00343.x

Santini, L., N. J. B. Isaac and G. F. Ficetola. 2018. "TetraDENSITY: a database of population density estimates in terrestrial vertebrates." *Global Ecology and Biogeography* 27: 787–791. https://doi.org/10.1111/geb.12756

Smith, A. and A. Lee. 1984. "The evolution of strategies for survival and reproduction in possums and gliders." In *Possums and Gliders*, edited by A. P. Smith and I. D. Hume, 17–33. Sydney, AU: Surrey Beatty and Sons.

Soria, C. D., M. Pacifici, M. Di Marco, S. M. Stephen and C. Rondinini. 2021. "COMBINE: a coalesced mammal database of intrinsic and extrinsic trait." *Ecology* 102: e03344. https://doi.org/10.1002/ecy.3344

Tidière, M., J.-M. Gaillard, V. Berger, D. W. H. Müller, L. B. Lackey, O. Gimenez, M. Clauss and J.-F. Lemaître. 2016. "Comparative analyses of longevity and senescence reveal variable survival benefits of living in zoos across mammals." *Scientific Reports* 6: 36361. https://doi.org/10.1038/srep36361

Todorov, O. S., S. P. Blomberg, A. Goswami, K. Sears, P. Drhlík, J. Peters and V. Weisbecker. 2021. "Testing hypotheses of marsupial brain size variation using phylogenetic multiple imputations and a Bayesian comparative framework." *Proceedings of the Royal Society B* 288: 20210394. https://doi.org/10.1098/rspb.2021.0394

Turbill, C., C. Bieber and T. Ruf. 2011. "Hibernation is associated with increased survival and the evolution of slow life histories among mammals." *Proceedings of the Royal Society B* 278: 3355–3363. https://doi.org/10.1098/rspb.2011.0190

Weisbecker, V., K. Ashwell and D. Fisher. 2013. "An improved body mass dataset for the study of marsupial brain size evolution." *Brain, Behavior and Evolution* 82: 81–82. https://doi.org/10.1159/000348647

Williams, S. E., J. VanDerWal, J. Isaac, L. P. Shoo, C. Storlie, S. Fox, E. E. Bolitho, C. Moritz, C. J. Hoskin and Y. M. Williams. 2010. "Distributions, life-history specialization, and phylogeny of the rain forest vertebrates in the Australian Wet Tropics." *Ecology* 91: 2493. https://doi.org/10.1890/09-1069.1

Wilman, H., J. Belmaker, J. Simpson, C. de la Rosa, M. M. Rivadeneira and W. Jetz. 2014. "EltonTraits 1.0: Species-level foraging attributes of the world's birds and mammals." *Ecology* 95: 2027. https://doi.org/10.1890/13-1917.1

Wilson, D. E. and R. A. Mittermeier. 2015. "Handbook of Mammals of the World, Vol. 5: Monotremes and Marsupials." Barcelona, ES: Lynx Edicions.
